# The First Report on the Complete Sequence Characterization of Bluetongue Virus Serotype 3 in the Republic of Korea

**DOI:** 10.3390/vetsci11010029

**Published:** 2024-01-11

**Authors:** Hyun-Jeong Kim, Jun-Gu Choi, Da-Seul Seong, Jong-Uk Jeong, Hye-Jung Kim, Sang-Won Park, Seung-Pil Yun, In-Soon Roh

**Affiliations:** 1 Division of Foreign Animal Disease, Animal and Plant Quarantine Agency, Gimcheon-si 39660, Republic of Korea; hjkim1945@gnu.ac.kr (H.-J.K.);; 2 Laboratory Animal Research Center, Central Scientific Instrumentation Facility, Gyeongsang National University, Jinju 52828, Republic of Korea; 3 Department of Pharmacology, Institute of Medical Sciences, College of Medicine, Gyeongsang National University, Jinju 52727, Republic of Korea; 4 Department of Convergence Medical Sciences, Gyeongsang National University Graduate School, Jinju 52727, Republic of Korea

**Keywords:** bluetongue virus, complete-genome sequencing, serotype 3, cattle, reassortment

## Abstract

**Simple Summary:**

Bluetongue (BT) is an economically significant disease in ruminants, transmitted worldwide by *Culicoides* midges, with the causative agent being the bluetongue virus (BTV). Traditionally prevalent in tropical regions, its geographic range is expanding due to climate change. Despite its global impact, research on the BTV in the Republic of Korea (ROK) is scarce. In this study, we aimed to assess BTV seroprevalence and conduct genetic analyses on isolates from South Korea. Out of 5824 cattle samples, 194 (3.33%) showed BTV antibodies, and 2 out of 1095 (0.18%) goat samples were positive. BTV RNA analyses of 422 high-risk area cattle samples revealed 51 (12.1%) positive cases. The virus was successfully isolated from one sample, identified as serotype 3 based on segment 2 analysis. This study presents the first full-genome sequence of a South Korean BTV serotype 3 isolate, serving as a global reference. These data aid in understanding phylogenetic relationships among BTV strains and recognizing reassortment events, enhancing our knowledge of this developing virus.

**Abstract:**

The bluetongue virus (BTV) is a significant animal pathogen with economic implications in the ruminant industry. Despite global reports on BTV detection and epidemiologic investigations, limited studies have focused on the virus in the ROK. In this study, BTV epidemiological research was conducted on blood samples from cattle and goat farms across nine regions during 2013–2014. The results showed that 3.33% of bovine blood samples (194/5824) and 0.19% of goat blood samples (2/1075) tested positive for BTV antibodies using ELISA. In Jeju-do, BTV RNA amplification occurred in 51 of 422 samples (12.1%) using real-time reverse transcription (RT-qPCR). The isolation of one sample revealed it as serotype 3, as indicated by the sequence of segments 2 (Seg-2) and 6 (Seg-6), associated with the eastern BTV topotype. However, based on Seg-1, -3, -4, -5, -7, -8, -9, and -10 analyses, the BTV-3/JJBB35 strain is more closely related to distinct BTV strains. These findings imply BTV circulation and that the Korean-isolated BTV might originate from Asian BTV strains due to multiple reassortment events. This study provides foundational data for ongoing BTV monitoring and disease-control policies in the ROK.

## 1. Introduction

Bluetongue (BT) is a non-contagious, arthropod-borne viral disease caused by the bluetongue virus (BTV) in ruminants. The disease predominantly affects sheep but impacts cattle, which serve as the primary reservoir due to their relatively prolonged viremia, playing a crucial role in the disease’s epidemiology [1]. The BTV is transmitted by *Culicoides* midges, and historically, it was confined to tropical and subtropical regions between the latitudes of 35° S and 40° N, mainly prevalent in Central Africa, America, Australia, and Southeast Asia [2]. However, outbreaks in Northern Europe over the past decade have highlighted the unexpected adaptability of the BTV to cooler climates [3,4], potentially influenced by climate change, international trade, globalization, and vector population density [4,5]. Recent studies have underscored the primary mode of BTV transmission through the wind-borne movement of *Culicoides* midges over substantial distances, notably observed in previously unaffected areas [4,6].

As a member of the *Orbivirus* genus in the *Reoviridae* family, the BTV contains 10 segments (Seg-1 to Seg-10) of double-stranded RNA (dsRNA) enclosed in a triple-layered capsid, encoding at least seven structural (VP1–VP7) and four non-structural proteins (NS1 to NS4) [1,3,7]. The segmented nature of its genome allows reassortment to occur in cases of double infection with different BTV strains in a single host. This process generates entirely new combinations of genome segments in progeny viruses, varying genetically from their parental strains. Reassortment can alter transmissibility, break down host species’ barriers, and increase virulence or pathogenicity [3,8].

The outer capsid layer of the BTV contains two proteins, VP2 and VP5, which are encoded by Seg-2 and Seg-6 genomes, respectively. In particular, VP2/Seg-2 exhibits significant variability among viral proteins and plays a crucial role in serum neutralization and serotype specificity [9,10,11,12]. Even within the same serotype, subtypes such as ‘eastern (e)’ and ‘western (w)’ have been further identified to reflect the geographic origin of the virus strains [9,10]. Recent studies based on full-genome sequence analyses have demonstrated the importance of reassortment in the emergence of viruses with novel serotypes [8,10].

There are 27 BTV serotypes recognized as notifiable diseases by the World Organisation for Animal Health (WOAH) [10]. In recent years, several novel BTVs, referred to as ‘atypical’ strains, have been identified. Presently, 36 BTV serotypes have been officially characterized [11,12]. Among BTV serotypes, BTV serotype 3 (BTV-3) exhibits at least two main clusters: The first comprises strains from Africa, the Mediterranean Basin, and North America as western topotypes (BTV-3w), and the second includes strains from Japan, India, and Australia as eastern topotypes (BTV-3e) [13]. BTV-3w, in particular, has recently been reported in Italy, Israel, Tunisia, the Netherlands, and the United Kingdom, among other locations. Bluetongue is considered a re-emerging disease in Europe and the Mediterranean basin [13,14,15,16,17].

Although BT is globally recognized as a transboundary and re-emerging disease, there have been relatively few reports of BT in Asia compared to Europe. The main reason is that sheep are not a major livestock species in most countries in Asia, so BTV, which is known to cause serious pathogenicity in ruminants, mainly sheep, is of lesser importance [18]. Consequently, except for BTV testing required for import quarantine, research on more prevalent diseases in cattle and pigs has been prioritized over research on BTV disease outbreaks.

In Asia, Malaysia and Indonesia began testing for the BTV in sheep quarantined from Europe, sparking a gradual expansion of BTV surveillance systems [18]. Since then, the BTV has been detected in India and Australia, as well as in the Republic of Korea’s (ROK) neighboring countries of Japan and China [19,20,21,22,23,24,25,26,27,28,29,30]. Despite reports of the BTV in its immediate neighbors, there have been no further reports of the BTV in the ROK other than a report of BTV-1 in 2015 and BTV antibody confirmation in dairy cattle in 2019 [30,31].

To address the lack of information about the BTV in the ROK due to insufficient research, this study was designed to search for evidence of BTV circulation and isolate new viruses from the blood of cattle and goats collected during 2013–2014. Subsequently, the isolated viruses underwent whole-genome analysis to enhance our understanding of the BTV in neighboring Asian regions.

## 2. Materials and Methods

### 2.1. Collection of Samples

To identify BTV infection in ruminants in the ROK, our surveillance system consisted of passive and active components. Passive surveillance involved reporting suspected BTV cases, although no tests were requested during the study period. Active surveillance comprised statistical and targeted approaches. The statistical surveillance system was introduced to determine antibody prevalence in domestic ruminants. The number of tests was based on the size of cattle herds in each region, with individuals randomly selected for testing.

In the case of the targeted surveillance system, in Jeju-do, located at the southernmost tip of the ROK and identified as a high-risk region for vector-borne diseases [32,33,34], additional tests were undertaken to detect BTV RNA. This measure was implemented to swiftly confirm disease outbreaks and detect the bluetongue virus. In total, 6919 blood samples, including 5824 from cattle and 1095 from goats (taken from 1350 cattle farms and 263 goat farms), were collected from 1613 ruminant farms spanning nine provinces between January and October 2013 and 2014. In Jeju-do, a total of 422 cattle blood samples were collected and tested for the BTV from June to November, the peak vector activity period.

### 2.2. Detection of BTV Antibodies

Serum samples were tested for antibodies against the BTV using an enzyme-linked immunosorbent assay (ELISA), i.e., the ID Screen^®^ Bluetongue Competition assay (ID VET, Montpellier, France), according to the manufacturer’s instructions. Based on the ELISAs’ cutoff values, tested samples with an inhibition percentage of <35% for bluetongue were considered positive.

### 2.3. RNA Extraction and Real-Time RT-PCR(RT-qPCR)

Total RNA was extracted from 200 μL of a whole-blood sample or supernatant of harvested cells using an RNeasy Mini Kit (Qiagen, Valencia, CA, USA) according to the manufacturer’s instructions. The final RNA elution of 50 μL was stored at −80 °C until usage. Primers and a probe for initial RT-qPCR screening described by Orrù et al. [35] were used to detect BTV segment 10. RT-qPCR was performed on a CFX96 Touch real-time PCR detection system (BIO-RAD, Hercules, CA, USA) using an AgPath-ID™ one-step RT-PCR kit (Thermo Fisher Scientific, Waltham, MA, USA).

### 2.4. Virus Isolation

Whole-blood samples that tested positive in RT-qPCR were subjected to isolation attempts in a KC cell line derived from Culicoides sonorensis, which underwent blind passage after initial inoculation. After blind passage in a KC cell culture, the cells were disrupted through sonication followed by centrifugation to eliminate cellular debris. The resulting supernatant was used to inoculate baby hamster kidney (BHK21) cells. Samples showing characteristic cytopathological changes within 7 days of infection underwent reverse transcription PCR (RT-PCR) with specific primers to confirm the isolation of BTV.

### 2.5. Sequencing and Phylogenetic Analysis

To obtain genomic data on all 10 segments, the isolated BTV strain from the blood sample underwent RT-PCR using specific primer pairs designed to amplify approximately 1 kb of overlapping segments across various virus serotypes (as listed in Table S1). The primers and RT-PCR conditions used for Seg-2 followed the methods outlined in a previous study [31]. For full-length sequence analysis, the 5′ and 3′ terminal sequences (untranslated regions (UTRs)) of BTV strains were determined using the full-length amplification cDNA (FLAC) method, as described by Maan et al. [36]. The amplified products were sent to Bionics (Seoul, ROK) for sequencing, and the complete full-length sequences were produced using the ChromasPro package (Ver 2.1.5, Technelysium Pty Ltd., Brisbane, QLD, Australia). The percent similarity among genetic sequences for the 10 BTV segments analyzed in this study was assessed using the information from BLAST programs (NCBI, Bethesda, MD, USA). Higher-identity sequences were aligned using BioEdit alignment editor v. 7.0.5 and were subjected to ClustalW Multiple alignment [37]. The phylogenetic trees of each BTV segment were constructed in accordance with the neighbor-joining method in Molecular Evolutionary Genetics Analysis (MEGA 7) software [38]. The sequences determined in this study were deposited in GenBank under accession numbers MG922835–MG922843 (BTV-1) and MG922844–MG922853 (BTV-3).

## 3. Results

### 3.1. Detection of BTV Antibodies in Cattle and Goats

ELISA-based antibody testing revealed that among the 5824 blood samples collected from cattle during 2013–2014, 194 samples (3.33%) tested positive for BTV antibodies. In the case of goat blood samples—1075 in total—only 2 samples (0.19%) were positive for BTV antibodies. Notably, among the nine regions, Jeju-do, situated in the southernmost part of the ROK, showed the highest antibody rate (25.5%) when assessing seroprevalence among cattle. Looking at the results by year, it was confirmed that in the case of bovine serum, the seropositivity rate was 6.37% (150/2354) in 2014, which was higher than the antibody positivity rate of 1.26% (44/3480) in 2013. Conversely, the antibody titer of BTV in goats remained consistently low overall (refer to Table 1 for detailed data). Unfortunately, the neutralizing antibody test aimed at determining the specific BTV serotype was no longer possible due to a lack of available serum samples.

### 3.2. Molecular Detection and BTV Isolation

A total of 422 bovine blood samples were gathered from 162 farms in Jeju-do, and BTV RNA detection was conducted using RT-qPCR. Out of these tests, 51 samples were confirmed positive for BTV (12.1%). Notably, despite the absence of clinical symptoms, we successfully isolated the virus from blood collected from a 39-month-old Korean native cow reared in Jeju-do in 2014. This isolated virus was denoted as JJBB35.

### 3.3. Sequence and Phylogenetic Analyses

**Figure 1:**
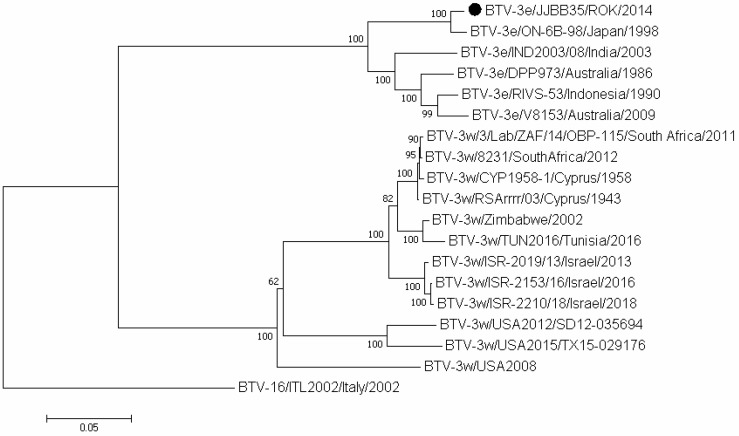
Phylogenetic analysis of Seg-2 of a Korean BTV-3/JJBB35 strain compared with globally published BTV strains. The percentage of replicate trees in which the associated taxa clustered together in the bootstrap test (1000 replicates) is shown next to the branches. Outgroup BTV-16 (ITL2002) was used for the phylogenetic tree of segment 2. The BTV-3/JJBB35 strain is marked with a black dot.

To confirm the serotype of the isolated strain JJBB35, the genome sequence was analyzed based on segment 2 (Seg-2), which was identified as serotype 3 (Figure 1). It had a total length of 2934 bp with an open reading frame (ORF) of 2837 bp. Based on this analysis, a BLAST search was performed in the NCBI database, and Table 2 shows BTV reference strains that exhibited high identity with each BTV-3/JJBB35 strain segment.

**Figure 2:**
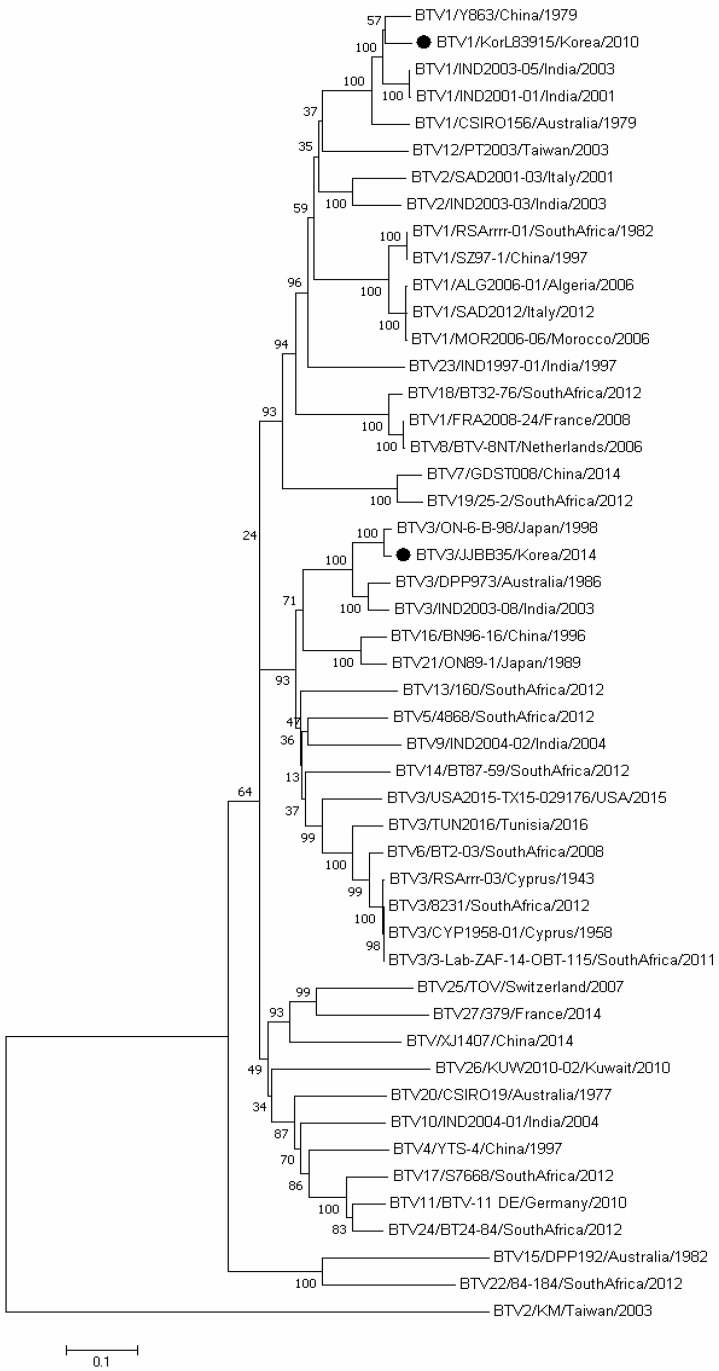
Phylogenetic analysis of Seg-6 of two Korean strains compared with globally published BTV sequences. The percentage of replicate trees in which the associated taxa clustered together in the bootstrap test (1000 replicates) is shown next to the branches. Korean isolates are marked with black dots.

**Figure 3:**
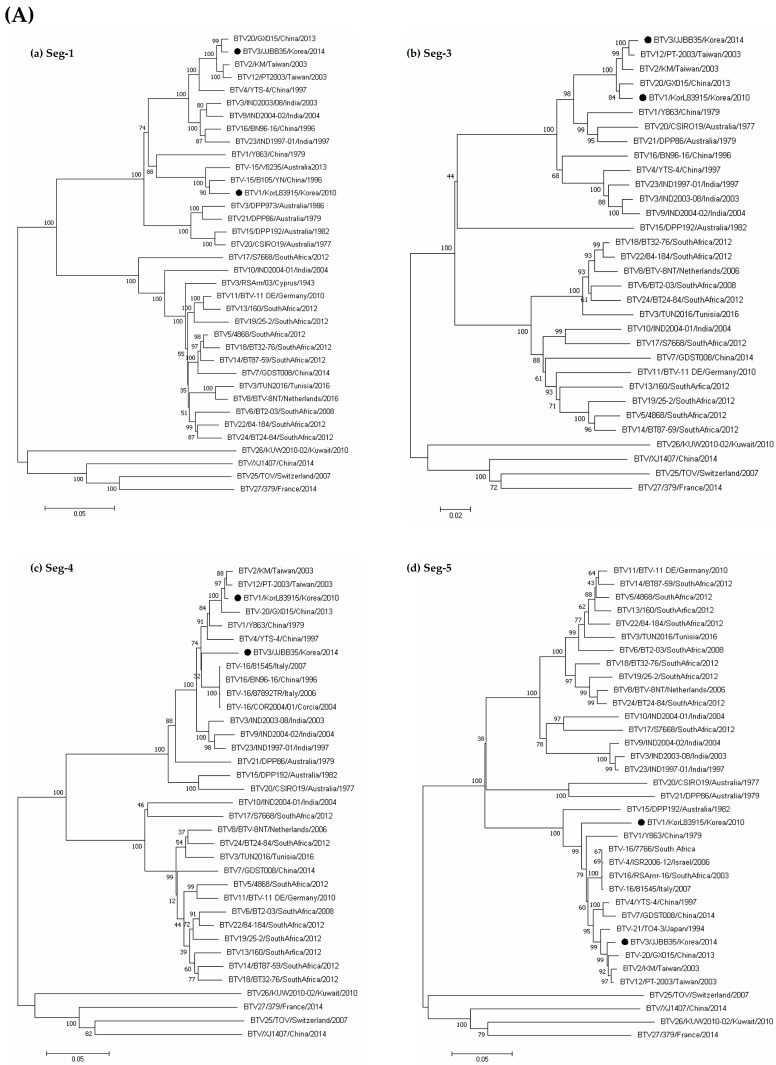

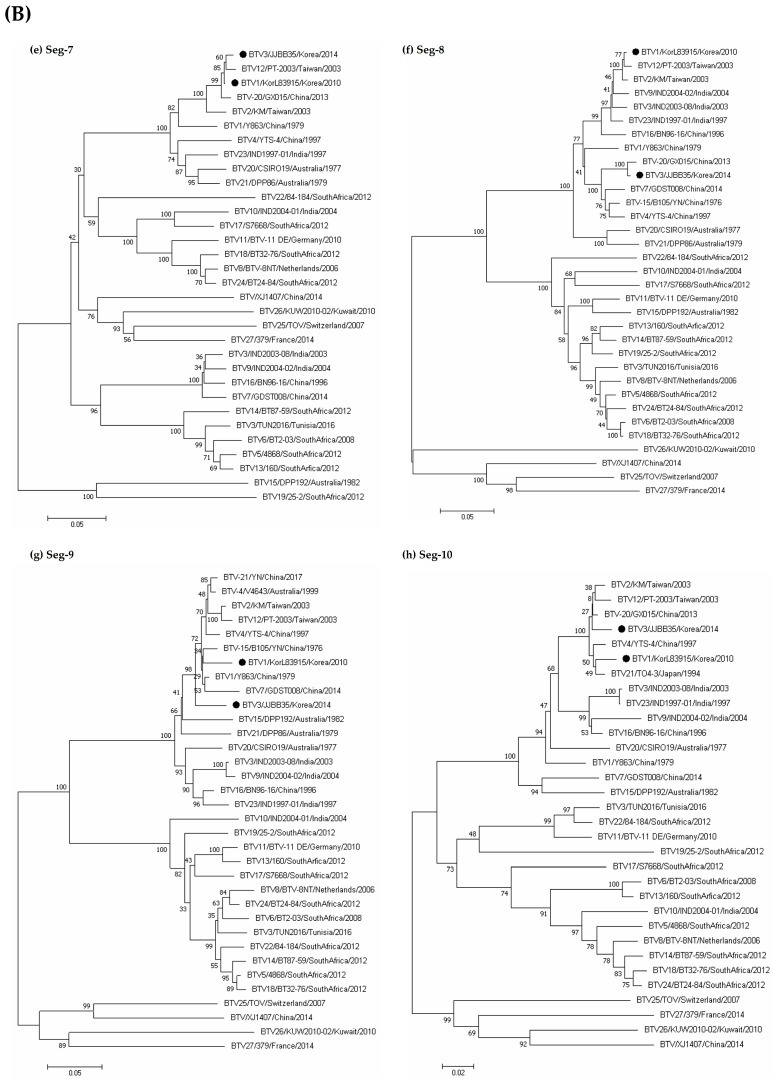
Phylogenetic trees based on the full-length nt sequences of (**A**) Seg-1, Seg-3, Seg-4, Seg-5, (**B**) Seg-7, Seg-8, Seg-9, and Seg-10 of Korean BTV strains (BTV-3/JJBB35 and BTV-1/KorL83915) compared with globally published BTV sequences. The percentage of replicate trees in which the associated taxa clustered together in the bootstrap test (1000 replicates) is shown next to the branches. Korean isolates are marked with black dots.

Comparisons of Korean BTV strains’ nt sequences with global BTV strains with available sequence data in GenBank were performed using a BLAST search (Table 2). The Seg-6 of the Korean BTV-3/JJBB35 strain exhibited a close relationship to the Japanese BTV-3 strain ON-6/B/98, with a maximum nt sequence similarity of 98.1%. Seg-1, Seg-8, and Seg-10 showed 99.1%, 99.0%, and 98.9% homology to the GX015 strain (BTV-20) from China, respectively (Table 2 and Figure 2). Seg-3 and Seg-5 exhibited a high homology with Taiwan’s PT strain, belonging to serotype 2, with homologies of 99.0% and 98.9%, respectively. Furthermore, Seg-4 displayed 95.9% homology with the BN96-16 strain (BTV-16) from China. Seg-7 and Seg-9 showed high homology with BTV strains belonging to BTV-1 (KorL83915 and Y863). This confirms that all segments of the BTV-3/JJBB35 strain exhibited high homology and clustering with isolates from Taiwan, China, India, and Australia within the eastern topotype. Most segments of the BTV-1/KorL83915 strain cluster with strains from China, Taiwan, and Japan, similar to the BTV-3/JJBB35 strain. However, Seg-5 clusters with Italy’s isolate (81545) within the western topotype, showing a 94.8% homology (Table 2 and Figure 3).

Terminal hexanucleotide sequences (5′-GTTAAA and ACTTAC-3′) are completely conserved among all segments of Korean BTV strains, including BTV-1/KorL83915 (Table S2).

## 4. Discussion

The BTV is one of the most devastating diseases in livestock, capable of causing significant economic losses. When it occurs, the most effective control measure is vaccination [39,40]. In this regard, epidemiologic information on the prevalence and serotype of the BTV can be crucial for vaccine application. While atypical bluetongue virus outbreaks have recently been increasing in Europe [41], there have been no reports of clinically symptomatic BT outbreaks in livestock in the ROK. Consequently, the BTV has not been well studied in the ROK. This study confirms the annual circulation of BTV infections in the ROK through antibody prevalence and BTV RNA detection. Additionally, this study is the first to report on the isolation and genetic analysis of BTV-3 in the ROK.

In this study, the presence of BTV RNA was determined in the blood of 422 cattle from Jeju-do, with 51 samples testing positive (12.1%). BTV-3/JJBB35 was isolated from one of these samples. NT sequence and phylogenetic analyses of Korean BTV strains revealed their close relationship with other Asian strains, including those from Japan, Taiwan, China, and other Asian countries. Comparing the Seg-2 complete-genome sequence of JJBB35 with eastern topotype strains from Japan (ON-6/B/98), India (IND2003-08), and Australia (DPP973/1986) showed nt sequence homologies of 98.3%, 90.0%, and 89.5%, respectively. Similarly, a comparative analysis of the Seg-2 amino acid (aa) sequence indicated homologies of 98.3%, 95.7%, and 93.8% to the same strains, as in the analysis of nt sequences, respectively. However, BTV-3 from the ROK showed low homologies of 69.8–70.9% for nt sequences and 74.2–74.9% for aa sequences with BTV-3 strains isolated from South Africa, Zimbabwe, Cyprus, Tunisia, and the United States, which are classified as western topotypes. As described in a previous study, the BTV1/KorL83915 strain underwent a full Seg-2 sequence analysis [31]. The results of the phylogenetic analysis confirmed that it was grouped with Asian BTV-1 isolates within the eastern topotype.

Based on the complete-genome sequencing and phylogenetic analysis of Seg-6, the BTV-3/JJBB35 strain also exhibited the highest homology (98.1%) with ON-6/B/98, a Japanese isolate. It clustered with BTV-3 strains from India (IND2003-08) and Australia (DPP973). In the case of the BTV-1/KorL83915 strain, another domestic isolate, the complete-genome sequencing and phylogenetic analysis of Seg-6 revealed a high homology with the Chinese isolate Y863 (93.8%) and the Indian isolates IND1992-02 and IND2001-01 (both 93.1%).

The results of the genetic analysis of two Korean BTV isolates showed that they are so-called reassortant, mainly mixed with various BTV strains isolated from neighboring Asian countries. The BTV-3/JJBB35 strain isolated in this study shared similarities with strains from Taiwan, China, India, and Australia, suggesting an origin in eastern Asia. However, a specific segment (Seg-5) in BTV-1/KorL83915 showed a connection with a BTV strain from Italy and South Africa, hinting at a different, western origin. Seg-5 encodes the non-structural protein 1 (NS1) gene, known for its impact on the virus’s efficiency in spreading. When reassortant strains possess this western NS1 gene, the virus’s ability to replicate is significantly affected, potentially influencing its strength and quantity [42]. Considering these results, although the BTV currently does not lead to clinical symptoms and is not pathogenic enough to be reported as a disease, there is always a potential risk that pathogenic viruses might be generated as the recombination process continues.

As a result of the BTV antibody test conducted in this study, 3.33% (194/5824) of cattle tested positive, with Jeju-do showing the highest rate at 25.5%, confirming once again that there were BTV infections nationwide. In the serological survey for BTV infection conducted by Hwang’s research team around the same time, serotypes BTV-1, -2, -3, -4, -7, -15, and -16 were identified using neutralizing antibody methods [30]. By combining the results of the BTV antibody test and viral RNA detection in this study with previous studies [30,31], it can be inferred that BTV infection in ruminants, particularly in cattle in the ROK, occurs frequently, leading to recurring infections among domestic cattle in the affected regions.

The primary route of BTV introduction into the country is through potential infections in imported animals [4,43]. According to quarantine statistics from the Animal and Plant Quarantine Agency regarding livestock imports, only 10 cattle were imported from 2012 to 2014: 3 in 2012, 3 in 2013, and 4 in 2014. Furthermore, all 10 animals were imported from the United States and Canada, which have no links to the BTV-3 strain isolated in this study. No abnormalities were found in the quarantine process, suggesting that this route is unlikely to be responsible for the domestic introduction of the BTV strain in question.

Another potential route for disease introduction could be associated with the movement of *Culicoides* spp., which is a disease-mediating vector. Recent modeling studies on *Culicoides* spp. have explored its modes of transmission. These studies indicate that *Culicoides* can cover significant distances by being carried in the wind (over 100 km), suggesting the potential introduction of new virus serotypes in previously unaffected areas [4,6]. This finding supports the notion that BTV strains in the ROK and neighboring countries like Japan, Taiwan, and China can influence one another and suggests that occurrences in the ROK might be attributed to foreign *Culicoides* transported by the wind from neighboring regions.

Based on the research data obtained to date, it is not clear how the BTV was introduced in the ROK or how it affects our neighbors. In addition, domestic research results obtained to date are insufficient to explain how domestic BTV strains are connected with neighboring Asian countries, even though the time gap is quite large. However, taken together, the data from this study confirm that BTV infections circulated in ruminants in the ROK. Moreover, we isolated a new BTV-3 strain and conducted whole-genome analyses on 10 genes, providing additional information on the BTV-3 eastern topotype that can be valuable for molecular epidemiological studies of the BTV. To our knowledge, BTV-3 was first isolated in the ROK, and this is the first time Korean isolates have been characterized through a whole-genome analysis. The data obtained in this study emphasize the need for further epidemiological studies and the consistent surveillance of the BTV in ruminants. This study also underscores the significance of genomic surveillance in monitoring the potential risk of new viruses emerging through the reassortment process with a novel pathogenicity.

Global warming, which also affects the ROK, contributes to the diversification of the type, number, and range of BTV-mediating *Culicoides*, which facilitates the expansion of BTV distribution and increases potential threats. Therefore, to gain a comprehensive understanding of the molecular epidemiology of causative agents for transmitted diseases like the BTV, which are prevalent in both the ROK and neighboring countries, collaborative research with adjacent nations is crucial. Furthermore, to better understand the BTV, continuous research on the types and distribution of *Culicoides*, a disease mediator (vector), along with broader serological monitoring and serotype analyses of the BTV, are essential.

## 5. Conclusions

In this study, we performed whole-genome sequencing for the newly isolated BTV-3/JJBB35 from bovine blood and analyzed all segments, except for Seg-2, for another Korean BTV isolate, BTV-1/KorL83915. These findings could serve as the foundation for ongoing studies on BTV epidemiology. Furthermore, conducting virus characterization through whole-genome analyses offers insights into virus origins and replication.

## Figures and Tables

**Table 1:** Distribution of the seroprevalence proportions of BTV in cattle and goats in the Republic of Korea (2013–2014).

Species	Regions	2013		2014		Total
		No. of Samples	No. of Positive Samples (%)	No. of Samples	No of Positive Samples (%)	
Cattle	Gyonggi-do	378	3 (0.79)	219	0 (0.0)	3/597 (0.5)
	Ganwon-do	746	2 (0.27)	140	0 (0.0)	2/886 (0.23)
	Chungcheongbuk-do	686	2 (0.29)	60	32 (53.3)	34/746 (4.56)
	Chungcheongnam-do	222	0 (0.0)	48	15 (31.3)	15/270 (5.56)
	Jeollabuk-do	170	0 (0.0)	60	0 (0.0)	0/230 (0.0)
	Jeollanam-do	469	1 (0.21)	392	1 (0.26)	2/861 (0.23)
	Gyeongsangbuk-do	309	0 (0.0)	564	1 (0.18)	1/873 (0/11)
	Gyeongsangnam-do	265	3 (1.13)	604	6 (0.99)	9/869 (1.04)
	Jeju-do	235	33 (14.0)	267	95 (35.6)	128/502 (25.5)
	Total	3480	44 (1.26)	2354	150 (6.37)	194/5834 (3.33)
Goat	Gyonggi-do	144	0 (0.0)	-	-	0/144 (0.0)
	Ganwon-do	58	0 (0.0)	-	-	0/58 (0.0)
	Chungcheongbuk-do	64	0 (0.0)	-	-	0/64 (0.0)
	Chungcheongnam-do	20	0 (0.0)	-	-	0/20 (0.0)
	Jeollabuk-do	25	0 (0.0)	-	-	0/25 (0.0)
	Jeollanam-do	100	1 (1.0)	60	0 (0.0)	1/160 (0.63)
	Gyeongsangbuk-do	32	0 (0.0)	56	0 (0.0)	0/88 (0.0)
	Gyeongsangnam-do	204	0 (0.0)	312	1 (0.32)	1/516 (0.19)
	Total	647	1 (0.15)	428	1 (0.23)	2/1075 (0.19)

**Table 2:** Comparison between nucleotide (nt) composition of Korean BTV strains and the closet global BTV strains.

Strain	Segment	Closest Sequence—nt Identity (%)
JJBB35	Seg-1	BTV-20/GX015/2013/China (99.1)
(BTV-3)	Seg-2	BTV-3/ON-6/B/98/Japan (98.3)
	Seg-3	BTV-12/PT/2003/Taiwan (99.0)
	Seg-4	BTV-16/BN96-16/1996/China (95.9)
	Seg-5	BTV-12/PT/2003/Taiwan (98.9)
	Seg-6	BTV-3/ON-6/B/98/Japan (98.1)
	Seg-7	BTV-1/KorL83915/2010/ROK (99.1)
	Seg-8	BTV-20/GX015/2013/China (99.0)
	Seg-9	BTV-1/Y863/1979/China (96.1)
	Seg-10	BTV-20/GX015/2013/China (98.9)
KorL83915	Seg-1	BTV-15/B105/YN/1976/China (97.5)
(BTV-1)	Seg-2	BTV-1/Kuala Lumpur/1987/Malaysia (93.9)
	Seg-3	BTV-20/GX015/2013/China * (97.8)
	Seg-4	BTV-12/PT/2003/Taiwan (99.1)
	Seg-5	BTV-16/81545/2007/Italy (94.8)
	Seg-6	BTV-1/Y863/1979/China (93.8)
	Seg-7	BTV-12/PT/2003/Taiwan (99.1)
	Seg-8	BTV-2/KM/2003/Taiwan (99.1)
	Seg-9	BTV-1/Y863/1979/China (96.5)
	Seg-10	BTV-4/YTS-4/1997/China (98.7)

* Strain sharing the same nt identity with other BTV strains belonging to BTV-2 and BTV-12.

## Data Availability

Data used in the current study can be found in NCBI nucleotide under the accession number indicated in the paper. Further inquiries can be directed to the corresponding authors.

## Supplementary Materials

The following supporting information can be downloaded at: https://www.mdpi.com/article/10.3390/vetsci11010029/s1, Table S1: List of primers used in the present study for the sequence of bluetongue virus.; Table S2: Characteristics of 10 segments of the Korean BTV strains.

## References

[1] Caporale M., Di Gialleonorado L., Janowicz A., Wilkie G., Shaw A., Savini G., Rijn P.A.V., Mertens P., Ventura M.D., Palmarini M. (2014). Virus and host factors affecting the clinical outcome of bluetongue virus infection. J. Virol..

[2] MacLachlan N.J., Osburn B.I. (2006). Impact of bluetongue virus infection on the international movement and trade of ruminants. J. Am. Vet. Med. Assoc..

[3] Nomikou K., Hughes J., Wash R., Kellam P., Breard E., Zientara S., Palmarini M., Biek R., Mertens P. (2015). Widespread reassortment shapes the evolution and epidemiology of bluetongue virus following European invasion. PLoS Pathog..

[4] Kelso J.K., Milne G.J. (2014). A spatial simulation model for the dispersal of the bluetongue vector *Culicoides brevitarsis* in Australia. PLoS ONE.

[5] Purse B.V., Mellor P.S., Rogers K.J., Samuel A.R., Mertens P.P., Baylis M. (2005). Climate change and the recent emergence of bluetongue in Europe. Nat. Rev. Microbiol..

[6] Jacquet S., Huber K., Pagès N., Talavera S., Burgin L.E., Carpenter S., Sanders C., Dicko A.H., Djerbal M., Goffredo M. (2016). Range expansion of the Bluetongue vector, *Culicoides imicola*, in continental France likely due to rare wind-transport events. Sci. Rep..

[7] Roy P., Knipe D.M., Howley P.M., Cohen J.I., Griffin D.E., Lamb R.A. (2007). Orbiviruses and their replicaion. Fields Virology.

[8] Shaw A.E., Ratinier M., Nunes S.F., Nomikou K., Caporale M., Golder M., Allan K., Hamers C., Hudelet P., Zientara S. (2013). Reassortment between two serologically unrelated bluetongue virus strains is flexible and can involve any genome segment. J. Virol..

[9] Maan N.S., Maan S., Belaganahalli M.N., Ostlund E.N., Johnson D.J., Nomikou K., Mertens P.C. (2012). Identification and differentiation of the twenty six bluetongue virus serotypes by RT-PCR amplification of the serotype-specific genome segment 2. PLoS ONE.

[10] WOAH Terrestrial Manual 2021 Chapter 3.1.3. Bluetongue (Infection with Bluetongue Virus). https://Woah.org/fileadmin/Home/eng/Health_standards/tahm/3.01.03_BLUETONGUE.pdf.

[11] Ries C., Sharav T., Tseren-Ochir E.O., Beer M., Hoffmann B. (2020). Putative novel serotypes ‘33’ and ‘35’ in clinically healthy small ruminants in Mongolia expand the group of atypical BTV. Viruses.

[12] Ries C., Vögtlin A., Hüssy D., Jandt T., Gobet H., Hilbe M., Burgener C., Schweizer L., Häfliger-Speiser S., Beer M. (2021). Putative novel atypical BTV serotype ‘36’ identified in small ruminants in Switzerland. Viruses.

[13] Golender N., Bumbarov V., Eldar A., Lorusso A., Kenigswald G., Varsano J.S., David D., Schainin S., Dagoni I., Gur I. (2020). Bluetongue Serotype 3 in Israel 2013–2018: Clinical Manifestations of the Disease and Molecular Characterization of Israeli Strains. Front. Vet. Sci..

[14] Cappai S., Rolesu S., Loi F., Liciardi M., Leone A., Marcacci M., Teodori L., Mangone I., Sghaier S., Portanti O. (2019). Western Bluetongue virus serotype 3 in Sardinia, diagnosis and characterization. Transbound. Emerg. Dis..

[15] Lorusso A., Sghaier S., Di Domenico M., Barbria M.E., Zaccaria G., Megdich A., Portanti O., Seliman I.B., Spedicato M., Pizzurro F. (2018). Analysis of bluetongue serotype 3 spread in Tunisia and discovery of a novel strain related to the bluetongue virus isolated from a commercial sheep pox vaccine. Infect. Genet. Evol..

[16] Holwerda M., Santman-Berends I.-M.G.A., Harders F., Engelsma M., Vloet R.-P.M., Dijkstra E., van Gennip R.-G.P., Mars M.H., Spierengurg M., Roos L. (2023). Emergence of bluetongue virus serotype 3 in the Netherlands in September 2023. bioRxiv.

[17] Gray A. (2023). UK confirms cases of bluetongue serotype-3. Vet Rec..

[18] Daniels P.W., Sendow I., Pritchard L.I., Sukarsih, Eaton B.T. (2004). Regional overview of bluetongue viruses in South-East Asia: Viruses, vectors and surveillance. Vet. Ital..

[19] Saminathan M., Singh K.P., Khorajiya J.H., Dinesh M., Vineetha S., Maity M., Rahman A.F., Misri J., Malik Y.S., Gupta V.K. (2020). An updated review on bluetongue virus: Epidemiology, pathobiology, and advances in diagnosis and control with special reference to India. Vet. Q..

[20] Boyle D.B., Ritchie R.A., Broz I., Walker P.J., Melville L., Flanagan D., Davis S., Hunt N., Weir R. (2014). Evolution of Bluetongue Virus Serotype 1 in Northern Australia over 30 Years. J. Virol..

[21] Goto Y., Yamaguchi O., Kubo M. (2004). Epidemiological observations on bluetongue in sheep and cattle in Japan. Vet. Ital..

[22] Kato T., Shirafuji H., Tanaka S., Sato M., Yamakawa M., Tsuda T., Yanase T. (2016). Bovine arboviruses in Culicoides biting midges and sentinel cattle in southern Japan from 2003 to 2013. Transbound Emerg. Dis..

[23] Shirafuji H., Yanase T., Kato T., Yamakawa M. (2012). Genetic and phylogenetic characterization of genome segments 2 and 6 of bluetongue virus isolates in Japan from 1985 to 2008. J. Gen. Virol..

[24] Miura Y., Inaba Y., Tsuda T., Tokuhisa S., Sato K., Akashi H. (1982). Seroepizootiological survey on bluetongue virus infection in cattle in Japan. Natl. Inst. Anim. Health Q..

[25] Yang H., Lv M., Sun M., Lin L., Kou M., Gao L., Liao D., Xiong H., He Y., Li H. (2016). Complete genome sequence of the first bluetongue virus serotype 7 isolate from China: Evidence for entry of African-lineage strains and reassortment between the introduced and native strains. Arch. Virol..

[26] Sun E.C., Huang L.P., Xu Q.Y., Wang H.X., Xue X.M., Lu P., Li W.J., Liu W., Bu Z.G., Wu D.L. (2016). Emergence of a novel bluetongue virus serotype, China 2014. Transbound. Emerg. Dis..

[27] Zhu J., Yang H., Li H., Xiao L., Wang J., Li N., Zhang N. (2013). Full-genome sequence of bluetongue virus serotype 1 (BTV-1) strain Y863, the first BTV-1 isolate of Eastern origin found in China. Genome Announc..

[28] Yang T., Liu N., Xu Q., Sun E., Qin Y., Zhao J., Feng Y., Wu D. (2012). Complete genomic sequence of bluetongue virus serotype 1 from China. J. Virol..

[29] Yang H., Zhu J., Li H., Xiao L., Wang J., Li N., Zhang N., Kirkland P.D. (2012). Full genome sequence of bluetongue virus serotype 4 from China. J. Virol..

[30] Hwang J.M., Kim J.G., Yeh J.Y. (2019). Serological evidence of bluetongue virus infection and serotype distribution in dairy cattle in South Korea. BMC Vet. Res..

[31] Seo H.J., Park J.Y., Cho Y.S., Cho I.S., Yeh J.Y. (2015). First report of Bluetongue virus isolation in the Republic of Korea and analysis of the complete coding sequence of the segment 2 gene. Virus Genes.

[32] Jeong D.-Y., Ragen P., Juan R., António A. (2015). Final Report of the Project on the Impact of Climate Change on Island and Coastal Biosphere Reserves. UNESCO Digital Library. https://unesdoc.unesco.org/ark:/48223/pf0000246972.

[33] Son W.-S. (2023). Climate Change and Tourism Sustainability in Jeju Island Landscape. Sustainability.

[34] Climate Change Jeju City. https://www.meteoblue.com/en/climate-change/jeju-city_south-korea_1846266.

[35] Orrù G., Ferrando M.L., Meloni M., Liciardi M., Savini G., de Santis P. (2006). Rapid detection and quantitation of bluetongue virus (BTV) using a molecular beacon fluorescent probe assay. J. Virol. Methods.

[36] Maan S., Rao S., Maan N.S., Anthony S.J., Attoui H., Attoui H., Samuel A.R., Mertens P.P.C. (2007). Rapid cDNA synthesis and sequencing techniques for the genetic study of bluetongue and other dsRNA viruses. J. Virol. Methods.

[37] Hall T.A. (1999). BioEdit: A user-friendly biological sequence alignment editor and analysis program for window 95/98/NT. Nucleic Acids Symp. Ser..

[38] Kumar S., Stecher G., Tamura K. (2016). MEGA7: Molecular Evolutionary Genetics Analysis version 7.0 for bigger datasets. Mol. Biol. Evol..

[39] Rojas J.M., Martín V., Sevilla N. (2021). Vaccination as a Strategy to Prevent Bluetongue Virus Vertical Transmission. Pathogens.

[40] Van Rijn P.A. (2019). Prospects of Next-Generation Vaccines for Bluetongue Front. Vet. Sci..

[41] Spedicato M., Compagni E.D., Caporale M., Teodor L., Leone A., Ancora M., Mangone I., Perletta F., Portanti O., Giallonardo F.D. (2022). Reemergence of an atypical bluetongue virus strain in goats, Sardinia, Italy. Res. Vet. Sci..

[42] Maan S., Maan N.S., Belaganahalli M.N., Rao P.P., Singh K.P., Hemadri D., Putty K., Kumar A., Batra K., Krishnajyothi Y. (2015). Full-Genome Sequencing as a Basis for Molecular Epidemiology Studies of Bluetongue Virus in India. PLoS ONE.

[43] Hornyák Á., Malik P., Marton S., Dóró R., Cadar D., Bányai K. (2015). Emergence of multireassortant bluetongue virus serotype 4 in Hungary. Infect. Genet. Evol..

