# Supplementary material for: The First Report on the Complete Sequence Characterization of Bluetongue Virus Serotype 3 in the Republic of Korea
Source: Vet Sci. 2024 Jan 11;11(1):29. doi: 10.3390/vetsci11010029 (PMC10821305; doi:10.3390/vetsci11010029)
Supplement: Supplementary file 1 [file vetsci-11-00029-s001.zip › vetsci-2734658-supplementary.pdf]

Supplementary Table 1. List of primers used in the present study for the sequence of bluetongue virus.

Primer	Nucleotide sequence(5′-3′)*	Target gene	Position	Size(bp)	Remarks
S1-1F	GTTAAAATGCAATGGTCG	Seg-1 (VP1)	1-18	1459	
S1-1459R	ATAACTARAGC TTRATCC		1441-1459		
S1-1167F	TGGGTGAAGAACGTGAG		1167-1183	1274	
S1-2440R	ACAGAATAHGGYGTCATC AT		2421-2440		
S1-1985F	TTAGRA CKA CAT TYGATG C		1985-2003	1359	
S1-3343R	GKCCRATC TCATCTTCTG		3325-3343		
S1-3074F	TTCGAGAACCATGATGCA		3074-3091	855	
S1-3944R	GTAAGTGTAATGCGGCG		3928-3944		
S1-530R	AGT CRG CCA TAA CYT GCA		513-530		For double checking terminal sequence
S1-3402F	AAG GAT GTY GTK ATG AGA GG		3402-3421		
P1 F	GTTAAAATAGTAGCGCG	Seg-2 (VP2)	1-24	1315	Reference [31]
P2 R	TGATTYARCYTATAYTCCCA		1296-1315		
P3 F	TTMGACCMACMTATACBCCYTA		1186-1207	915	
P4 R	CGYGATTCMTAY ACWYCTCT		2082-2102		
P5 F	ATWTWTGGCGAGAGCATYGTR		2001-2021	939	
P6 R	GTAAGTMTRATAGTRCGCG		2922-2940		
S3-1F	GTT AAA TTT CCG TAG CCA	Seg-3 (VP3)	1-18	1456	
S3-1456R	CGC GAA TCR ATR CCA CA		1440-1456		
S3-1350F	ACR GGA ACA GGR TAT AAT GG		1350-1369	1423	
S3-2772R	GTA AGT GTR TTY CCG CTG C		2754-2772		
S3-485R	GTT AAA TTT CCG TAG CCA				For double checking terminal sequence
S3-2316F	GAT GAT GAY GAY TGG GTA AG				
S4-1F	GTT AAA ACA TGC CTG AGC	Seg-4 (VP4)	1-18	1459	
S4-1459R	ATA TCT GGG TAC TTC CAC A		1441-1459		
S4-918F	TTR ATY AAA CAT AGG ATA CC		918-937	1064	
S4-1981R	GTA AGT TKT ACA TGC CC		1965-1981		
S4-615R	TAC GTA RRT CRC CGC ATC				For double checking terminal sequence
S4-1405F	GAG TTY TRG ATG GYA ATG G				
S5-1F	GTT AAA AAA GTT CTC TAG TTG GC	Seg-5 (NS1)	1-23	1176	
S5-1176R	ACY GAA TYA TTW CCC ACA TG		1157-1176		
S5-746F	AAY AGR TAT GCT TCG ATG G		746-764	1018	
S5-1763R	GTA AGT TGA AAA GTT CTA GTA GAG T		1739-1763		
S5-534R	GCRATTTGRCGATATCTYG				For double checking terminal sequence
S5-1227F	GGRGATCATTAYTATACAA				
S6-1F	GTT AAA AAG ATC CCY AYG AT	Seg-6 (VP5)	1-20	1160	
S6-1160R	ACA TCA TCA GAR TCC CAT GG		1141-1160		
S6-659F	GGK ATG CAR GAR GAR GCG AT		659-678	979	
S6-1637R	GTA AGT AKA AGT CCC GRG A		1619-1637		

S6-434R	CATTCATRAATTGATACACCTC				For double checking terminal sequence
S6-1110F	CAACCACAAATGCACATA				
S7-1F	GTT AAA AAT CTM KAG AGA TGG A	Seg-7 (VP7)	1-22	1154	
S7-1154R	GTA AGT GTA ATC YHA GAG ACG		1134-1154		
S7-296R	ACWCCAATTGTMGCCATATG				For double checking terminal sequence
S7-829F	TATAGCTTYAGAGATCAYAC				
S8-1F	GTT AAA AAA TCC TTG AGT CAT G	Seg-8 (NS2)	1-22	1125	
S8-1125R	GTA AGT GTA AAA TCC CCC		1108-1125		
S8-370R	CATCACWCCATTATGTTGYA				For double checking terminal sequence
S8-574F	CGYTGGATGGATGAWGA				
S9-1F	GTT AAA AAA TCG CAT ATG TC	Seg-9(VP6)	1-20	1052	
S9-1052R	GTA AGT GTR AAA TCG CMC TA		1033-1052		
S9-469R	TTCGTACCGTATTTAGATTC				For double checking terminal sequence
S9-762F	GTCATGAGTCAAAAGAARCT				
S10-1F	GTT AAA AAG TGT CGC TG	Seg-10 (NS3, NS3a)	1-17	822	
S10-822R	GTG AGT GTR TAG YGY CG		806-822		
S10-288R	CTCACATCATCACGAAAC				For double checking terminal sequence
S10-610F	GGAAGTKATGAAGAAACAAT				

* Abbreviations are for a mixed-base code. Y = C, T; K = G, T; R = A, G; W = A, T; H= A, C, T; R=A, G; M=A, C

Supplementary Table 2. Characteristics of 10 segments of the Korean BTV strains.

BTV serotype/ Strain	Segment /Protein (Size)	Open Reading Frame (nt)	G+C Content (%)	Size of Protein (aa)	5′ terminal sequences*	3′ terminal sequences*	GenBank accession number
BTV-1/ Korl83915	Seg-1/VP1 (3944)	12-3917	42.29	1302	**GTTAAA**ATGCAATGGTCGC	AGCGCCGCATTAC**ACTTAC**	MG922835
	Seg-2/VP2 (2944)	22-2907	41.88	960	**GTTAAA**ATAGTAGCGCGAT	CGCGCACTATCAG**ACTTAC**	KC153300
	Seg-3/VP3 (2772)	18-2720	43.94	901	**GTTAAA**TTTCCGTAGCCAT	GCAGCGGAACCAC**ACTTAC**	MG922836
	Seg-4/VP4 (1981	9-1940	43.06	644	**GTTAAA**ACATGCCTGAGCC	GGGGGCATGCAAA**ACTTAC**	MG922837
	Seg-5/NS1 (1763)	35-1690	43.51	552	**GTTAAA**AAAGTTCTCTAGT	ACTAGAACTTTTCA**ACTTAC**	MG922838
	Seg-6/VP5 (1635)	26-1603	43.55	526	**GTTAAA**AAGTGTACCCTTA	GGGAAGCACTTAC**ACTTAC**	MG922839
	Seg-7/VP7 (1154)	18-1064	46.97	349	**GTTAAA**AATCTCTAGAGAT	TCTCTGAGATTAC**ACTTAC**	MG922840
	Seg-8/NS2 (1125)	20-1081	44.98	354	**GTTAAA**AAATCCTTGAGTC	GGGGGGATTTTAC**ACTTAC**	MG922841
	Seg-9/VP6 (1052)	16-1005	47.05	330	**GTTAAA**AAATCGCATATGT	AGTGCGATTTTAC**ACTTAC**	MG922842
	Seg-10/ NS3, NS3a (822)	20-706<br>59-706	45.62	229<br>216	**GTTAAA**AAGTGTCGCTGCC	CGCGGCGCTACAC**ACTTAC**	MG922843
BTV-3/ JJBB35	Seg-1/VP1 (3944)	12-3917	42.70	1302	**GTTAAA**ATGCAATGGTCGC	AGCGCCGCATTAC**ACTTAC**	MG922844
	Seg-2/VP2 (2934)	21-2897	41.27	959	**GTTAAA**AACGTTGCCCGGA	TGGGCACGTTTTC**ACTTAC**	MG922845
	Seg-3/VP3 (2772)	18-2720	43.98	901	**GTTAAA**TTTCCGTAGCTAT	GCAGCGGGAATAC**ACTTAC**	MG922846

Seg-4/VP4 (1981)	9-1940	43.11	644	**<u>GTTAAA</u>**ACATGCCTGAGCC	GGGGGCATGTAAA**<u>ACTTAC</u>**	MG922847
Seg-5/NS1 (1763)	35-1690	42.77	552	**<u>GTTAAA</u>**AAAGTTCTCTAGT	CTAGAACTTTTCA**<u>ACTTAC</u>**	MG922848
Seg-6/VP5 (1637)	29-1606	44.47	526	**<u>GTTAAA</u>**AAGTTCCCTACGA	TCTCGGGACTTAT**<u>ACTTAC</u>**	MG922849
Seg-7/VP7 (1154)	18-1064	47.05	349	**<u>GTTAAA</u>**AATCTCTAGAGAT	TCTCTAAGATTAC**<u>ACTTAC</u>**	MG922850
Seg-8/NS2 (1125)	20-1081	44.36	354	**<u>GTTAAA</u>**AAATCCTTGAGTC	GGGGGGATTTTAC**<u>ACTTAC</u>**	MG922851
Seg-9/VP6 (1052)	16-1005	47.43	330	**<u>GTTAAA</u>**AAATCGCATATGT	AGCGCGATTTTAC**<u>ACTTAC</u>**	MG922852
Seg-10/ NS3, NS3a (822)	20-706<br>59-706	45.74	229<br>216	**<u>GTTAAA</u>**AAGTGTCGCTGCC	CGCGGCGCTATAC**<u>ACTTAC</u>**	MG922853

* Conserved hexanucleotide sequences in the 5′ and 3′ termini are shown in bold and underlined.
